# Selection for increased quorum-sensing cooperation in *Pseudomonas aeruginosa* through the shut-down of a drug resistance pump

**DOI:** 10.1038/s41396-018-0205-y

**Published:** 2018-06-20

**Authors:** Ron D. Oshri, Keren S. Zrihen, Itzhak Shner, Shira Omer Bendori, Avigdor Eldar

**Affiliations:** School of Molecular Cell Biology and Biotechnology, Faculty of Life Sciences, Tel-Aviv University, Tel-Aviv, Israel

## Abstract

The opportunistic pathogen *Pseudomonas aeruginosa* employs a hierarchical quorum-sensing network to regulate virulence factor production that cooperatively benefit the population at a cost to the individual. It has been argued that the evolution of a cooperative mutant in a quorum sensing-suppressed population would be hampered through its exploitation by neighboring non-mutant cells. It remains unclear whether mechanisms which overcome this exploitation exist. Here we investigate the regain of quorum-sensing cooperation by evolving a mutant of the *lasR* master quorum-sensing regulator. The mutant regained partial cooperative growth through null mutations in *mexT*, which codes for an activator of the MexEF-OprN multidrug-resistant pump. We find that these mutations enhance cooperative growth in both the *lasR* mutant and wild-type backgrounds through the activation of the RhlIR system. We show that the regain of cooperation in *mexT* mutants is mediated by the reduction in MexEF-OprN activity, whereas an additional source of private benefit is mostly *mexEF*-*oprN*-independent. Finally, we show that addition of antibiotics for which resistance is mediated by MexEF-OprN prevents the selection of increased cooperation at sub-MIC concentrations. MexT, therefore, not only links private and public goods, but also exposes conflicts between selection for antibiotic resistance and enhanced cooperation.

## Introduction

Bacteria engage in altruistic cooperation by secreting a variety of costly molecules which modify the environment to the benefit of the bacterial community. Such public goods molecules include enzymes, toxins, antibiotics, and surfactants [1–3]. Public goods secretion is often regulated by quorum-sensing cell–cell signaling, which depends on the release of a signal molecule and its detection by a cognate receptor [4, 5]. Quorum sensing leads to a density-dependent response, which improves the efficiency of public goods utilization [6–8].

Regulation of secretion by quorum sensing is crucial for the lifestyle of the opportunistic pathogen *Pseudomonas aeruginosa*. This species has a complex network of quorum-sensing systems, consisting of the LasIR and RhlIR systems whose signal molecules belong to the Acyl-homoserine-lactone family and the Pseudomonas quinolone signal (PQS) system, which codes for several quinolone signal molecules [9]. These systems are organized hierarchically with the Las system at the top tier, regulating the activation of the Rhl and PQS systems, which also cross-regulate in a complex manner [10–13]. The quorum-sensing network of *P. aeruginosa* controls the secretion of a variety of virulence factors including all secreted proteases [12, 14, 15].

An intrinsic problem with quorum sensing-dependent cooperative secretion is its susceptibility to exploitation by quorum-sensing mutants, which do not secrete the public goods, but enjoy their benefits [5]. Such cheater mutants gain an increased fitness over the wild-type strain and are therefore able to invade the population and reduce the level of cooperation or even lead to a population collapse [16, 17]. Indeed, *lasR* mutants of *P. aeruginosa* have been shown to evolve when the wild-type bacteria were grown in a medium whose only carbon source is casein, which requires quorum-sensing-dependent secretion of proteases for its digestion [18, 19]. Interestingly, *lasR* mutants also emerge during chronic infections, though the question of whether they arise by cheating is yet unresolved [20–22].

A related question is whether cooperative variants would be able to invade a community dominated by cheater genotypes. This general question has been previously explored in the fruiting body formation process of *Myxococcus xanthus* [23, 24], yeast secretion of invertase [25] and *P. aeruginosa* siderophore secretion [26]. A similar problem arises in the context of evolution of resistance to anti-quorum-sensing drugs, where a resistant mutant will be counter-selected thorough exploitation by its non-mutated neighbors [5, 27, 28]. This theoretical claim was demonstrated in a simple setting for *P. aeruginosa*, by reversing the role of a *lasR* mutant (which represented the inhibited wild-type) and the wild-type (which represented the inhibitor-resistant mutant) [29, 30]. These experiments do not rule out the evolution of other compensatory mutations, or the evolution of resistance in structured population [31, 32].

In the case of *P. aeruginosa*, the effect of a *lasR* deletion (or LasR inhibition by drugs) on public goods secretion may be partially complemented by a mutation resulting in a Las-independent activation of the Rhl system. Decoupling of the Las and Rhl systems has been demonstrated to occur under certain environmental conditions [10, 33–35]. Therefore, mutations that will extend the range of conditions in which decoupling occurs, are likely to emerge. As in other cases, it is unclear whether such mutant will be able to overcome exploitation by neighboring ancestral cells. Interestingly, recent analysis of clinically evolved *lasR* mutant isolates, indicates that some have partially reactivated their Rhl system [20].

Two decades ago, the Iglewski group evolved a *lasR* mutant in casein medium. They obtained a suppressive mutation that led to overproduction of the Rhl signal molecule and allowed the cells to regain their cooperative growth [36]. Here, we revisit this experiment with modern sequencing tools to identify the causal mutations that shape the regain of cooperation. We show that this regain can be attributed to an inactivation of the *mexT* transcriptional regulator, which activates the expression of the multidrug-resistant pump MexEF-OprN. This regulatory mutation has two main contributions to fitness; it leads to a regain of cooperation through induction of the Rhl system via a MexEF-OprN-dependent mechanism, and privately benefit the cells via a mostly MexEF-OprN-independent mechanism. As the MexEF-OprN pump is crucial for antibiotic resistance, this mechanism leads to a conflict between the evolutionary regain of cooperation and antibiotic resistance.

## Materials and methods

### Bacterial strains and plasmids

Bacterial strains used in this study are listed in Supplementary Tables S1, S2. Strains were either acquired from other laboratories (Greenberg, Banin, Köhler) or arose by evolution. The *mexT*^−^;Δ*lasR* deletion strain was constructed by introducing the *lasR* deletion into strain AEA325 as previously described [37]. All promoter reporter plasmids were cloned into the pPROBE plasmid (kindly provided by the Banin laboratory) [38], using the primers described in Supplementary Table S3 with standard cloning methods. Constitutive BFP and GFP reporter plasmids were constructed using the backbone of pMRP9-1 [39], under the control of the constitutive P_A1/04/03_ promoter [40]. Both BFP and GFP were synthetically designed as *P. aeruginosa* codon-optimized variants and obtained from GENEWIZ (Plainfield, USA). The amino-acid sequences of GFP and BFP are of GFPmut2, and Azurite, respectively.

### Growth conditions

Routine growth of cultures was done in lysogeny broth (LB) medium. Antibiotic was added when needed, at the following concentrations, *Escherichia coli*, 100 μg/mL ampicillin; *P. aeruginosa*, 300 μg/mL carbenicillin. For the relevant experiments (Fig. 4), chloramphenicol (Cm) stock at a concentration of 50 μg/mL in ethanol was diluted into the various final concentrations. We found that ethanol (Cm carrier) levels had a significant effect on growth in casein and therefore carrier levels were kept constant when comparing growth at different Cm concentrations. Casamino-Acids (CAA) and casein media were made as previously reported [18]. 0.5% CAA medium: M8 minimal medium (5 g/L Na_2_HPO_4_*2H_2_O, 3 g/L KH_2_PO_4_, 0.5 g/L NaCl, 1 mM MgSO_4_, 100 μM CaCl_2_) and 5 g/L Acid-hydrolyzed casein (Difco), adjusted to pH = 7.4. 1% casein medium: M8 minimal medium and 10 g/L casein sodium salt from bovine milk (Difco), adjusted to pH = 7.4.

### Evolution of Δ*lasR* in casein

Fresh colonies of *P. aeruginosa* PAO1 Δ*lasR* strain (AEA102) were inoculated into 2 mL of 0.5% CAA medium and were cultured for 16 h at 37 °C with agitation. The following morning, OD_600_ of cultures was usually 1–2. The cultures were diluted to an OD_600_ of 0.1 into 1% casein medium and were left at 37 °C with agitation. Significant opacity was observed 32–37 days post incubation. Cells were incubated for 3 more days after the increase in opacity prior to isolation of mutants.

### Evolution of wild-type PAO1 (*mexT*^*+*^) in casein

Initial culturing was performed as above. Every 24 h, cultures were diluted into three new test-tubes containing casein medium with a dilution factor of 5, 10, 20. The test-tube with the highest dilution that showed significant growth was used in the next day to propagate the culture. Evolutionary improvement or loss of proteolytic activity were assayed every 4 days by comparing colony halo size on skim-milk plates for ~ 100 isolates (see next paragraph).

### Qualitative halo estimates on skim-milk plates

Cultures were diluted onto LB plates and incubated for 16 h at 37 °C. The following morning, 100 colonies were picked and spotted on skim-milk plates (with wild-type (AEA101), and Δ*lasR* (AEA102) as controls). The plates were incubated for 16 h at 30 °C and the following morning colonies were screened for quorum-sensing activity (growth and proteolytic activity) by comparing the size of the formed halo with that of the controls.

### Elastase assay

Elastase activity was quantified by a modification of a procedure described previously [36]. In brief, bacterial cultures were incubated with shaking at 37 °C for 12 h in 0.5% CAA medium. Ten mg of Elastin-Congo Red (Sigma) and 1.01 mL of reaction buffer (0.05 M Tris-HCl buffer, pH 7.0, 0.5 mM CaCl_2_) were added to 15 mL glass tubes. After centrifugation of the culture, 10 μl of supernatant was added to the glass tubes and the mixture was incubated at 37 °C for 4 h. The reaction was terminated by adding 100 μL of 0.12 M EDTA (pH 8.0). After centrifugation of the slurry, the absorbance at 495 nm of the supernatant was measured with a spectrophotometer zeroed on a control Elastin-Congo Red sample incubated without enzyme. As absorbance of *mexT*^*+*^ strains was lower than that of the blank, the absorbance value of all strains was corrected by subtracting the mean absorbance level of the strain with lowest absorbance level.

### Relative growth and competition measurements in casein

Relevant strains were transformed using pUB-GFP plasmid (Supplementary Table S2). Transformed strains were streaked from frozen stocks onto selective agar plates. After 16 h incubation at 37 °C, a single colony was picked and inoculated into 2 mL of a 0.5% CAA medium supplemented with carbenicillin. The colonies were cultured for 16 h at 37 °C with agitation. The following morning, OD_600_ of cells was measured (usually 1–2) and cultures were diluted to an OD_600_ of 0.1 into 2 mL of 1% casein medium. Prior to incubation, a sample from each culture was diluted into phosphate-buffered saline and cell count per unit time was determined using flow cytometer measurements of events expressing GFP. Cultures were then incubated at 37 °C with agitation for the indicated times. At each time point, cell counts were again measured by flow cytometry. Relative growth was calculated as the ratio of cell counts after and before incubation.

For each competition assay, relevant pairs of strains transformed with either the pUB-GFP or the pUB-BFP plasmids (Table S1), were streaked from frozen stocks onto selective agar plates and grown separately as described above. Strains were then diluted to an OD_600_ of 0.1 (unless otherwise stated) and mixed to the required relative frequencies. At time zero and at later indicated time points, flow cytometry was used to identify and quantify the number of BFP and GFP expressing cells. Each competition experiment was performed with switched markers and results shown are averages over multiple experiments, half with one marker combination and half with the other. It was clear from the experiments that the BFP plasmid had a slightly higher cost than the GFP plasmid (Fig. S4). Relative fitness of a focal strain over its co-cultured strain was calculated by dividing the relative frequency of the strains after incubation to that prior to incubation.

### Gene expression measurements in CAA and casein media

For CAA gene expression experiments, overnight cultures of the relevant strains were diluted to an OD_600_ of 0.0005 into 96-well plate containing 0.5% CAA medium supplemented with 300 μg/mL carbenicillin. Each well contained 100 μL CAA medium and 120 μL of mineral oil. The plate was incubated in a plate reader (2030 multilabel reader from PerkinElmer Victor X3) up to 72 h and wells were measured for OD_600_ and GFP levels every 15–20 minutes. Different repeats of the same experiment (both within and between days) were aligned by setting the time to + 3 h when their optical density read was at 0.1. To allow for calculation of means and standard deviations, readouts were interpolated into constant time-steps, the dense measurement sampling ensures that interpolation does not affect the results. For casein medium, strains carrying the P_*lasB*_-GFP reporter were grown overnight in CAA medium and then diluted to an OD_600_ of 0.1 into casein medium. GFP expression measurements were taken at the indicated time points using flow cytometry.

### Flow cytometry

All samples were run in a Gallios flow cytometer (Beckman-Coulter) equipped with four lasers (405 nm, 488 nm co-linear with 561 nm, 638 nm). The emission filters used were: BFP – 450/50, GFP – 525/40. All samples were run at the “low” acquisition rate to reduce intra-sample variability. Events were discriminated upon the forward-scatter parameter. For each run, at least 100,000 cells were counted, and the total events analyzed was such that minority population was never below 1000 events.

### Next-generation genomic sequencing and analysis

A total of nine strains were sequenced using deep sequencing. For each strain, an isolated colony was picked from an LB plate and inoculated into LB broth. The cultures were incubated for 16 h as outlined previously, and DNA was harvested with the Promega genomic DNA purification kit. Sequencing was done using the Technion sequencing services. All strains were pooled and ran on a single lane of a HighSeq illumina sequencer with a read length of 50 bp. Average coverage of each strain was ~90 ×. Analysis was done with the help of Professor Rotem Sorek, from the Weizmann Institute of Science, using a program that was developed at his Laboratory.

## Results

### Directed evolution of Δ*lasR* and wild-type PAO1 strains in casein medium lead to reproducible consecutive mutations in the genes *psdR* and *mexT*

It has been previously shown that growth in minimal medium with casein as a sole carbon source requires the quorum sensing-dependent secretion of proteases [18, 36]. We wondered whether a quorum-sensing mutant would be able to evolve cooperative growth in casein medium, despite possible exploitation by the non-evolved cells. To this aim, we inoculated four parallel lines with ~ 10^7^ cells of a Δ*lasR* PAO1 strain (strain AEA102, Table S1). As no growth was initially observed, test-tubes were incubated until any signs of growth would appear. As a control, we also evolved a wild-type PAO1 strain (strain AEA101, Table S1), following cycles of growth and dilutions similarly to what was previously published [18, 19] (methods).

Strikingly, we found that both the Δ*lasR* and wild-type genetic backgrounds evolved increased level of proteolytic activity. First, all four Δ*lasR*-containing test-tubes became opaque after 32–37 days of incubation, indicating increased casein hydrolysis activity and growth. The directed evolution experiments were terminated at that stage (methods) and isolates were picked from each replicate. The proteolytic activity of isolates was then assessed by their ability to form a degradation halo on skim-milk plates. We found that > 60% of the cells in each evolved population showed a substantial increase in their halo size compared with the halo of their Δ*lasR* parental strain. Second, we found that after several cycles of growth and dilution, the growth of the wild-type cells improved. Examination of isolates from the 7^th^ cycle revealed a high number ( > 50%) of isolates with increased halo size compared with the halo of their parental wild-type strain.

To identify the genetic basis for the regain of proteolytic activity and ability to invade the population, we chose seven isolates with high proteolytic activity from three of the Δ*lasR* evolved lines and one isolate from the evolved wild-type line for deep sequencing, alongside their parental strains. We also sent for sequencing an additional wild-type PAO1 strain (AEA325, Table S1), which displayed an increased level of proteolytic activity compared with the one which we used to initiate directed evolution (Table 1). Strikingly, we found that all evolved isolates but one, contained mutations in two genes compared to their respective parental strains; *psdR* and *mexT*. Multiple different mutations in these two genes were observed, including in strains obtained from the same evolved test-tube. Two isolates from the same test-tube contained the same *psdR* mutation, but different mutations in *mexT*, suggesting that the *psdR* mutation arose first. Notably, the highly proteolytic PAO1 strain (strain AEA325, Table S1) carried only a single mutation in the *mexT* gene compared with the wild-type strain we used in our directed evolution experiment, which displayed weaker proteolytic activity (AEA101, Table S1).

**Table 1:** Whole-genome sequencing results of various strains in comparison with their parental strain

			*psdR*		*mexT*			
Strain	Ancestor	Tube (Day)^a^	Allele	Effect	Allele	Effect	Additional genes affected	Cm^R^
AEA936	Δ*lasR*	4(20)	T149A	L → Q			PA1097 (*fleQ*)	*−* ^c^
AEA934	Δ*lasR*	4(20)	T149A	L → Q	G827A	G → D	PA3457^b^	*−*
AEA937	Δ*lasR*	4(20)	T149A	L → Q	G827A	G → D	PA0580 (*ygjD*)	*−*
AEA938	Δ*lasR*	4(20)	T149A	L → Q	G827A	G → D	PA3457^b^, PA2094^b^	*−*
AEA933	Δ*lasR*	2(37)	G3A	M1I (Loss of start codon)	T722A	L → Q		*−*
AEA941	Δ*lasR*	3(37)	A469C	T → P	T insertion at 547	Frame-shift	*psdR-dppA3 intergenic*	*−*
AEA942	Δ*lasR*	3(37)	A469C	T → P	T368C	L → P	*psdR-dppA3 intergenic*	*−*
AEA935	WT		G41A	R → H	C983A	P → Q	*psdR-dppA3 intergenic*	*−*
AEA325^d^					C712A	P → T		*−*

Shown are the specific mutations in the genes *psdR* and *mexT* and their consequences in the protein sequence. Additional genes which acquired mutations are also shown. The last column indicates whether the strains can grow in 100 μg/mL of chloramphenicol

^a^The day the sample was taken is in parenthesis

^b^Low coverage area/mixed reads—possible artifact

^c^ + and − Indicate whether the cell grew or did not grow on 100 μg/mL of chloramphenicol. A level in which the ancestors Δ*lasR* and wild-type strain did grow

^d^Kindly provided by the Banin lab

In addition to mutations in *psdR* and *mexT*, further mutations were found in other loci (Table 1). Two mutations were found in the intergenic region downstream of *psdR* and upstream of the *dppA* gene. These mutations are most likely functionally related to *psdR*. Other mutated genes appeared only in single isolates. One isolate did not carry a mutation in *mexT*, but rather in the gene *fleQ* [41]. We did not further characterize this mutation.

With a focus on *psdR* and *mexT*, we Sanger-sequenced those loci for 13 randomly chosen isolates from a single evolved Δ*lasR* line (Table 2). We found that 4 out of 13 isolates had wild-type alleles in both loci. All other nine strains contained one of four different mutations in *psdR*, including one nonsense mutation. Mutations in *mexT* were only found in a *psdR* mutant background. Different *mexT* mutations, including a large deletion, were found with the same *psdR* mutation. Altogether, the deep sequencing and Sanger sequencing results support a model in which *psdR* mutations have evolved first, followed by mutations in *mexT*. The selection of multiple different mutations in each of the genes, as well as the nature of some of the mutations (e.g., frameshifts, nonsense and start codon elimination, Tables 1, 2), suggests that they resulted in loss of function of the corresponding genes.

**Table 2:** Genotyping of *mexT* and *psdR* of 13 blindly chosen isolates from a single evolved line of the Δ*lasR* strain

Halo size phenotype	Number of isolates	*psdR* allele	Effect	*mexT* allele	Effect	Cm^R^
Ancestral	4	Wild-type		Wild-type		+
Ancestral	1	C209T	Q → Stop	Wild-type		+
Ancestral	1	A529C	T → P	Wild-type		+
Ancestral	1	A389C	V → A	Wild-type		+
Evolved	4	R431C	Y → S	G502A	E → K	−
Evolved	1	R431C	Y → S	A176C	D → A	−
Evolved	1	R431C	Y → S	Δ (51-165)	Truncation	−

The first column indicates the halo size on skim-milk; Ancestral (small) or evolved (large). The last column indicates whether the strains can grow in 100 μg/mL of chloramphenicol

PsdR is a negative regulator of genes involved in the intake and degradation of short peptides [42]. Recently, it was shown that growth in casein medium leads to strong selection for *psdR *null mutations in both a cooperator and an evolved *lasR* mutant [43]. It was also shown that the *psdR* mutation does not contribute directly to the secretion of proteases and is selected for its associated private benefit. We therefore focused on understanding the role of *mexT* in the evolution of regained cooperation.

MexT is a transcriptional regulator of a variety of genes [44]. This regulatory function includes the activation of the *mexEF-oprN* operon, which codes for an RND family multidrug-resistance pump critical for resistance to various substances, including the antibiotic chloramphenicol (Cm) [45, 46]. Loss of MexT activity should therefore directly lead to reduction in mexEF-oprN expression, which will result in Cm sensitivity. Previous works have identified significant diversity of *mexT* and *mexEF-oprN* alleles in PAO1 “wild-type” strains [47]. We therefore refer to all strains below as either *mexT*^*+*^ or *mexT*^*−*^ and avoid using the term “wild-type”.

**Fig. 1:**
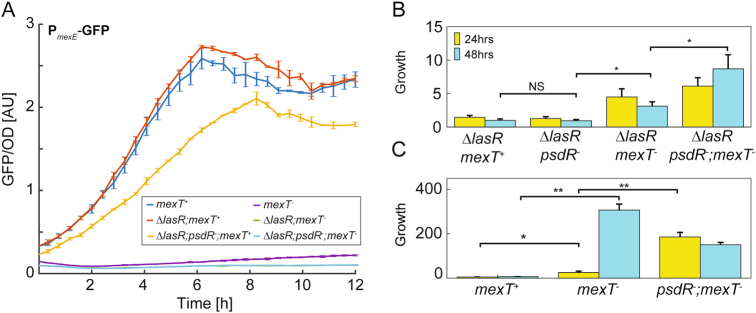
Characteristics of evolved mutants. **a** Activity of a GFP transcriptional reporter for the *mexE* promoter as a function of time. Activity is measured as fluorescence level divided by optical density. Results are shown for six genotypes grown in casamino acid medium; *mexT*^+^ (blue), Δ*lasR;mexT*^+^ (red), Δ*lasR;psdR*^−^;*mexT*^+^ (orange), *mexT*^−^ (purple); Δ*lasR; mexT*^*−*^ (green) and Δ*lasR;psdR*^−^; *mexT*^−^ (cyan). **b**, **c** Growth in casein medium of seven relevant genotypes. Growth was measured as the ratio between cell counts after and before the indicated incubation intervals using flow cytometry. Shown are results for **b** Δ*lasR* background strains inoculated at an initial optical density of 0.1, **c** *lasR*^*+*^ background strains, inoculated at an initial optical density of 0.01. Error bars represent standard error of three or more independent repeats on different days. Exact genotypes are provided in Supp. Tables S1, S2. NS—non significant. *—*p* value < 0.05, **—*p* value < 10^−3^. (color figure online)

The MexEF-OprN pump was shown to reduce the Rhl and PQS quorum-sensing activity [44, 48, 49], most likely through the export of HHQ (4-hydroxy-2-heptylquinoline), which serves both as a direct signal and an intermediate in the production of the PQS signal molecule [50]. To determine the effect of the *mexT* mutations on *mexEF-oprN* expression, we transformed the ancestral *mexT*^*+*^ and Δ*lasR;mexT*^*+*^ strains, as well as several evolved isolates, with a GFP transcriptional reporter plasmid for the *mexEF-oprN* operon expression (methods). In agreement with a loss-of-function effect of *mexT*, the GFP expression level of the *mexT* mutant strains was significantly lower than that of the strains coding for the *mexT* wild-type allele when grown in casamino-acids (Fig. 1a, *p* < 10^−3^ for all comparisons of the same genetic background, two sample *t* test for average of all samples from the last 2 h of sampling, *N* = 4 for each comparison).

Consistently, we also found that all isolates that coded for a mutated *mexT* became sensitive to Cm at a concentration of 100 μg/mL, to which the strains coding for the *mexT* wild-type allele were resistant (Tables 1, 2). Notably, the only evolved Δ*lasR* strain, which did not acquire a *mexT* mutation, but rather an *fleQ* mutation, also showed low Cm resistance (Table 1), suggesting that regain of cooperation in this strain is also mediated by MexEF-OprN inactivation. This is an unknown regulatory link, but we did not further characterize it in this work.

### Growth in casein reflects the functions of the *mexT* and *psdR* mutations

To better estimate the contribution of each mutation to cooperative growth in casein medium, we assayed the ability of each strain to clonally grow in casein. Generally, we found that Δ*lasR*-derived strains grew significantly slower than *lasR*^*+*^-derived strains when inoculated at the same initial density (Fig. 1b, Fig. S1). As cooperative growth depends on initial density [7, 25], we compensated for this growth difference by growing *lasR*^*+*^-derived strains from an initial inoculation, which is a factor of 10 lower than that of the Δ*lasR*-derived strains (Fig. 1c). In agreement with their qualitative effect on halo size (Fig. S2), we found that the *psdR*^*−*^ allele did not significantly increase growth of the Δ*lasR* mutant over an incubation period of 24 or 48 h (*p* > 0.6, two sampled *t* test). In contrast, the *mexT*^*−*^ allele showed a significant increase in growth (*p* < 0.02 after 48 h, two sampled *t* test, *N* = 12). The two mutations synergistically interacted to further increase growth in a *psdR*^*−*^*;mexT*^*−*^ double mutant strain (Fig. 1b, *p* < 0.03 two sampled *t* test, *N* = 12). Similar results, but with higher growth rate, were obtained with the *lasR*^*+*^ background (Fig. 1c).

### The m*exT* mutation has a strong effect on protease activity and other Rhl-dependent genes in Δ*lasR* and *lasR*^*+*^ backgrounds

**Fig. 2:**
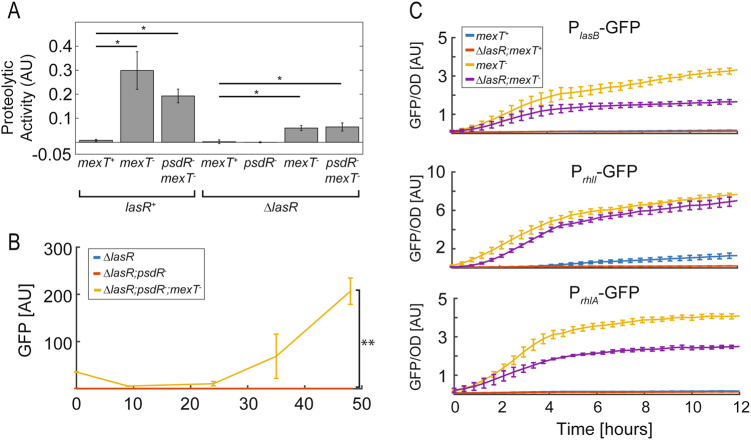
Proteolytic activity and gene expression of Rhl-dependent reporters in various genotypes and conditions. **a** Proteolytic activity was measured using an elastin-congo red assay for each of the genotypes (methods). **b** GFP expression average of single cells as measured using a P_*lasB*_-GFP reporter during growth in casein medium. Results are shown for three genotypes; Δ*lasR* (blue, overlapped by red), Δ*lasR;psdR*^*−*^ (red) and Δ*lasR;psdR*^*−*^*;mexT*^*−*^ (orange). Results were obtained using flow cytometry. **c** GFP per optical density as a function of time of GFP transcriptional reporters for *lasB* (top panel), *rhlA* (middle panel), and *rhlI* (bottom panel). Results in each panel are shown for four genotypes; *mexT*^*+*^ (light blue), Δ*lasR* (red), *mexT*^−^ (orange), and Δ*lasR; mexT*^−^(magenta). Error bars represent standard error of three or more independent repeats on different days. Exact genotypes are provided in Supplementary Tables S1, S2. *—*p* value < 0.05, **—*p* value < 2 × 10^−3^. (color figure online)

We wanted to verify that the *mexT*^*−*^ alleles lead to increased protease activity and a corresponding increase in protease gene expression in the *lasR*^*+*^ and Δ*lasR* mutant backgrounds. To this aim, we measured the protease activity of the various strains when grown in minimal medium using the elastin-congo red proteolysis assay (Fig. 2a). In addition, we compared halo sizes of all strains on skim-milk plates (Fig. S2). In both assays the *mexT*^*−*^ allele dramatically increased proteolytic activity, whereas the *psdR*^*−*^ allele had a negligible effect.

Next, we monitored protease expression in casein by measuring gene expression of the major protease gene, *lasB*. We transformed the Δ*lasR*, Δ*lasR;psdR*^*−*^, and Δ*lasR;psdR*^*−*^*;mexT*^*−*^ strains with a transcriptional GFP reporter for *lasB* and measured its activity during growth in casein using flow cytometry (Fig. 2b, methods). We found that the Δ*lasR*, and Δ*lasR;psdR*^*−*^ strains showed no *lasB* reporter expression throughout the experiment. In contrast, GFP expression was observed for the Δ*lasR;psdR*^*−*^*;mexT*^*−*^ strain (*p* < 2×10^−3^ for increased response compared with Δ*lasR* at 48 h, two sampled *t* test, *N* = 6). This expression was high upon initial inoculation into casein medium, reduced and then increased again during growth, in a manner corresponding to quorum sensing-dependent regulation. This observation further supports the role of the *mexT* null mutation in the evolution of cooperative protease secretion and suggests that the Rhl system, which is known to directly regulate *lasB* [15], became active in the Δ*lasR;psdR*^*−*^*;mexT*^*−*^ strain.

Previous works found that MexT activity has a negative impact on various Rhl-related behaviors in a *lasR*^*+*^ genetic background [44, 48]. To assess the relative effect of the *mexT*^*−*^ alleles on *lasR*^*+*^ and Δ*lasR* backgrounds, we monitored gene expression of the Rhl-dependent genes *lasB*, *rhlA,* and *rhlI*, using GFP transcriptional reporter plasmids during growth in casamino-acids medium (Fig. 2c, methods). For all the reporters, the *mexT*^*−*^ alleles had a dominant effect on gene expression, much stronger than that of the *lasR* deletion. The main effect of the Δ*lasR* allele was to delay the onset of activation of the Rhl reporters, with a relatively weak effect on their final levels. These results indicate that MexT impacts Rhl activity in a manner which is independent of LasR.

The observed effect of MexT on Rhl activity agrees with previous reports on the interaction between the MexEF-OprN pump, the PQS system and the Rhl system [10, 48, 50]. However, these results were obtained using a *lasR*^+^ background. As LasR is known to regulate the PQS receptor gene (*pqsR*) [51–53], it could be suggested that *lasR* deletion may eliminate or decrease PQS response to the *mexT*^*−*^ alleles. To verify that *mexT* affects PQS activity in a Δ*lasR* background, we measured the activity of a *pqsA* GFP transcriptional reporter in all four strain combinations of the *lasR* and *mexT* alleles (Fig. S3). We found that the *mexT*^*+*^ allele showed weak *pqsA* expression in both *lasR*^*+*^ and Δ*lasR* backgrounds. The *mexT*^*−*^ allele dramatically increased expression in both backgrounds (*p* < 10^−5^ in both cases, *N* = 10) but was twofold stronger in the *lasR*^*+*^ background (*p* < 10^−5^, *N* = 10). This indicates that *lasR* deletion, while affecting PQS response, does not eliminate the dependence on MexT activity.

### The *mexT*^*−*^ allele enhances cooperation through inactivation of MexEF-OprN, but increases private benefit in a MexEF-OprN-independent manner

A notable problem in the evolutionary regain of cooperative behaviors is their susceptibility to exploitation by ancestral non-cooperative strains [24, 26]. Specifically, for a *mexT*^*−*^ strain to invade into its ancestor *mexT*^*+*^ strain, its direct fitness should be increased irrespective of its ability to increase public goods secretion.

Previous work on MexT has ascribed the increase in Rhl activity primarily to the inactivation of the MexEF-OprN pump [54]. This suggests that the increase in public goods production observed in our evolutionary experiments could have also arisen through mutations to the *mexEF-oprN*. Nevertheless, all cooperation-enhancing mutations we identified were in the *mexT* gene, despite its small target size (~ 1 kb) compared with the *mexEF-oprN* operon (~ 6 kb). This led us to suspect that null mutation of *mexT* may mediate a private benefit to the cells in a *mexEF-oprN*-independent manner.

**Fig. 3:**
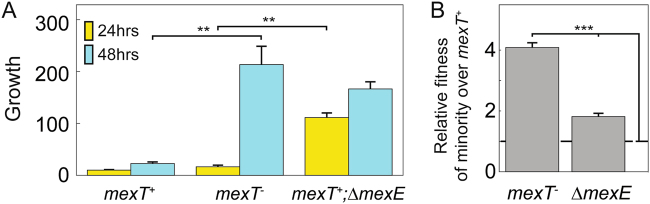
Growth **a** and selection in co-culture **b** of different *mex* variants. **a** Growth after 24 and 48 h of the efflux inactive strains *mexT*^−^*;mexE*^*+*^, *mexT*^*+*^;Δ*mexE*, and the efflux active *mexT*^*+*^*;mexE*^*+*^ strain. Growth is defined as in Fig. 1a, b. Averages and standard errors are calculated for multiple repeats performed on three separate days. **b** The strains *mexT*^*−*^*;mexE*^*+*^ (AEA1174) and *mexT*^*+*^;Δ*mexE* (AEA1176) were separately co-cultured as minorities with the efflux active strain *mexT*^*+*^*;mexE*^*+*^ (AEA1175). Shown is the relative fitness of each minority strain over the majority strain. Relative fitness is calculated by dividing the relative frequency of two co-cultured strains at the end of growth (after 48 h) by their relative initial frequency in the co-culture. A value of 1 indicates no fitness advantage. Initial frequency of the efflux inactive strains was set to ~ 5%. Co-cultured strains coded for a GFP and BFP constitutive reporters. Results are the summary of 18 co-culture experiments for each co-cultured pair, in half of the repeats the BFP was expressed in the majority strain and in half it was expressed in the minority strain. **a**, **b** Error bars represent the standard error of the mean. ** *p* value < 10^−3^, *** *p* value < 10^−6^. **b** Significance is also with respect to deviation from a value of one (no fitness difference). (color figure online)

In order to assess this hypothesis, we obtained an isogenic set of *mexT*^*−*^,*mexT*^*+*^ and *mexT*^*+*^;Δ*mexE* PAO1 strains (strains AEA1174, AEA1175, AEA1176, Table S1) [48]. We found that both the *mexT*^*−*^ and *mexT*^*+*^;Δ*mexE* strains grew much better than the isogenic *mexT*^*+*^ strain in casein medium (Fig. 3a, *p* < 10^−3^ for *mexT*^*−*^ over *mexT*^*+*^ after 48 h and for *mexT*^*+*^;Δ*mexE* over *mexT*^*+*^ after 24 h, two sample *t* test, *N* = 10). Interestingly, the *mexT*^*+*^;Δ*mexE* strain grew significantly faster than the *mexT*^*−*^ strain (*p* < 10^−5^ after 24 h, two sample *t* test, *N* = 10).

Next, we performed competition experiments between a majority of the proteolytically inactive *mexT*^*+*^ strain and a minority of either one of the proteolytically active strains (*mexT*^*−*^ or *mexT*^*+*^;Δ*mexE*) separately. (Fig. 3b, methods). We found that the *mexT*^*−*^ strain was able to increase its frequency by a factor of ~ 4 after 2 days of growth (*p* < 10^−10^, *t* test, *N* = 26). This validated the evolutionary trajectory obtained in the evolution experiments and demonstrated the strong private benefit to the mutant strain. In contrast, the frequency of the *mexT*^*+*^;Δ*mexE* strain increased only by a factor of 1.7 over two days of incubation (*p* < 10^−7^, *t* test, *N* = 26), suggesting that this mutation harbors a smaller private benefit to the cells than the *mexT*^*−*^ null allele (*p* < 10^−10^, *t* test). These results confirmed our hypothesis that a *mexT*^*−*^ null allele provides a strong private benefit to its carrier. The difference between the invasions of the two *mex* mutants suggests that much of this private benefit is independent of MexEF-OprN activity (see discussion).

### The adaptive advantage of *mexT* null alleles is eliminated in the presence of chloramphenicol

**Fig. 4:**
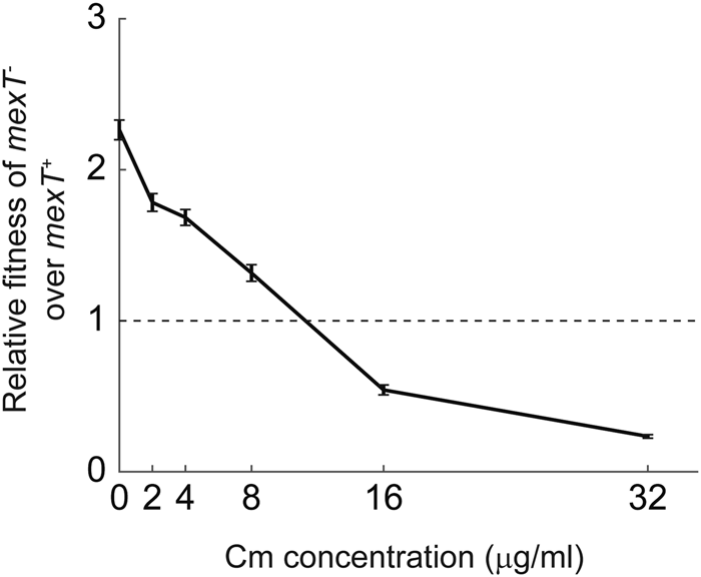
Relative fitness of a minority *mexT*^*−*^ (~ 5%, strain AEA1174) in a majority of *mexT*^*+*^ (strain AEA1175) after 48 h of growth in casein medium as a function of different concentrations of Chloramphenicol. All samples (including the no antibiotic sample) have the same level of ethanol carrier. Relative fitness is defined as in Fig. 3 caption. Error bars represent the standard error of the mean of six biological repeats, three with the minority marked with BFP and three with the minority marked with GFP. See Fig. S4 for separate graphs of the two markers.

Finally, the selection for a *mexT*^*−*^ null allele in an environment requiring quorum-sensing-dependent cooperation is opposite to the selection imposed by antibiotics for which resistance is mediated by the MexEF-OprN pump. This implies that selection for antibiotic resistance would prevent the regain of quorum-sensing activity and cooperation through inactivation of the MexEF-OprN pump. To further study this effect, we repeated the casein medium competition experiment between a minority of *mexT*^*−*^ strain and a majority of *mexT*^*+*^ strain in the presence of varying concentrations of Cm (Fig. 4). We found that the *mexT*^*−*^ relative fitness over the *mexT*^*+*^ strain was reduced with increased levels of Cm, becoming negative for Cm concentration larger than ~ 10 μg/mL. This concentration is substantially lower than the observed MIC of Cm for the *mexT*^*−*^ strain, which we found to be ~ 64 μg/mL (methods).

## Discussion

Evolutionary regain of cooperation is hampered by the ability of non-mutant cells to exploit the evolved cooperator [24, 26]. In the context of the evolutionary regain of quorum-sensing cooperation, exploitation is accompanied by the additional constrain imposed by the low levels of signaling molecules produced by the small minority of the evolved quorum-sensing cooperators [29, 30]. These limitations were proposed to improve the effectiveness of anti-quorum-sensing drugs [28]. Here, we found that a *lasR* deletion mutant, has evolved increased cooperation in casein medium through null mutations in *mexT*. Similar selection also occurred in a wild-type, *lasR*^*+*^, background. The *mexT* mutations arose after the selection of mutations in the *psdR* gene. *psdR* null mutants were previously shown to increase peptide utilization in *P. aeruginosa* [43].

The roles of MexT and the MexEF-OprN pump in the regulation of quorum-sensing activity have been analyzed in several previous works [44, 48, 49]. In addition, null mutations in *mexT* or *mexEF-oprN* are selected during clinical infections [47, 55] and lab culturing [47], leading to significant allelic diversity in these genes in clinical isolates and the PAO1 lab strain [45]. Despite the extensive literature on the subject, the interplay between the effects of *mexT* and *mexEF-oprN* on social and asocial sources of selection were not previously studied.

We found that the *mexT* null mutations increased the level of cooperative growth in both the Δ*lasR* and *lasR*^*+*^ backgrounds (Fig. 1). This increase is correlated with altered expression of Rhl-dependent genes in the *mexT*^*−*^ mutants (Fig. 2). We note, however, that the changes in gene expression in casamino acid medium (Fig. 2b–d), do not perfectly match those observed for growth in casein (Fig. 1a, b). Although both gene expression and growth results displayed a strong dependence on allelic variation in *mexT*, the effects of variation in the *lasR* locus had a smaller effect in the gene expression assays than their effect on growth in casein. This may stem from the relative dependence of other secreted proteases on LasR and RhlR or from the different growth medium used in the two experiments.

To better understand the interplay between private and cooperative effects of the *mexT*^*−*^ allele, we compared the cooperative growth and competitive fitness of the *mexT*^*−*^ and *mexT*^*+*^*;*Δ*mexE* strains (Fig. 3). Although both strains showed increased clonal growth and a competitive advantage over the *mexT*^*+*^ strain in casein medium, they differed in the relative levels of these two traits. The *mexT*^*−*^ strain showed a higher competitive advantage over the *mexT*^*+*^ strain than that of the *mexT*^*+*^*;*Δ*mexE* strain (explaining its selection, Fig. 3b). In contrast, the *mexT*^*+*^*;*Δ*mexE* strain had an increased level of clonal growth (Fig. 3a). It was previously shown that the *mexT*^*−*^ strain does not show any significant expression of the *mexEF-oprN* pump [56]. Therefore, the effects on both cooperative growth and competitive advantage, most likely arise through a MexEF-OprN-independent activity of MexT. A previous work identified several MexEF-OprN-independent functions of MexT, including the repression of the type III secretion systems, pyocyanin production, and surface attachment [54]. It is unclear whether the expression of these or other MexT-dependent traits could explain the observed differences.

We note that the *mexE* deletion was also beneficial to the cells, though to a lesser degree. This indicates that private benefit is also achieved via inactivation of the pump. This benefit may stem from several alternative sources. First, the MexEF-OprN pump activity was shown to affect proton gradient across the cell membrane, which may have adverse effects on growth [57]. Second, the private benefit may be mediated by PQS-related activities. Finally, the effect may be directly related to Rhl-associated private goods (e.g., cyanide resistance [58]) or to some level of privatization of exoprotease activity, which will lead to selection for exoprotease-secreting cells at low density, as was previously shown for yeast invertase secretion [25].

It is surprising that two mutations accumulated in the same cell during the evolution of the Δ*lasR* mutant despite the lack of apparent growth over the > 4 weeks of incubation. An initial population death phase in which *psdR* null alleles were selected for their reduced death rate may partially explain this evolutionary trajectory. The reduced population size would have allowed for a larger number of cell divisions over which the emergent *mexT* mutant alleles could have subsequently increase their frequency. Initially, the selection of the *mexT* mutants may depend mostly on their private benefit. Public goods secretion later leads to further growth and selection. Private and public benefits may therefore synergistically contribute to selection dynamics.

Pleiotropic genetic effects that link private and cooperative benefits have been previously observed in *P. aeruginosa* quorum-sensing [19, 58] and other cooperating microorganisms [59, 60]. In general, these pleiotropic couplings are expected to allow the reintroduction of cooperation into a cheater dominated population [23, 24] and slow down the emergence of cheaters in cooperator populations [59]. On a longer time-scale, pleiotropy on its own would not preclude the emergence of cheaters, as further mutations may uncouple the cooperative and private traits [61]. Specifically, in our case, while the initial evolution of the wild-type (*mexT*^*+*^) led to increased private and cooperative benefits through the effects of the *psdR* and *mexT* null alleles, further selection led to the emergence of *lasR* cheater mutants [18, 43]. In other cases, such as pyoverdine based cooperation, lack of pleiotropic dependence leads to a complete block on the evolutionary regain of cooperation [26].

Previous works on the evolution of quorum-sensing cooperation in casein and other protein-based media did not identify *mexT* as a locus affecting cooperation. This is most likely because a *mexT*^*−*^ allele was already fixed in the original genetic background of the starting cultures. We have verified this specifically with the authors of a previous work, which identified mutations in the *psdR* locus ([43], K. Asfahal, private communication). The presence of the *mexT*^*+*^ allele in some PAO1 strains (including the one used by us for directed evolution) and more so in the Δ*lasR* strain, where some of its phenotypes are masked, further demonstrates the pervasiveness of this allelic variation and the need to verify its status when considering quorum-sensing mutant phenotype [47].

Finally, RND family multidrug-resistant pumps have a crucial effect on the quorum-sensing network of *P. aeruginosa*. In addition to the MexEF-OprN pump discussed here, the MexAB-OprM pump exports the Las signal, 3-oxo-C12 homoserine lactone [62, 63], and its overexpression was shown to reduce quorum-sensing activity [64]. MexAB-OprM is also regulated by quorum-sensing, forming a feedback on quorum-sensing activity [49]. RND family of multidrug-resistance pumps appear to play a similar role in other Gram-negative bacteria with quorum-sensing systems, which utilize long-chained homoserine lactone autoinducers [65]. In all these cases, strong activation of the pumps may be detrimental to quorum-sensing activity, but necessary for antibiotic resistance. Antibiotic selection is therefore expected to greatly modify the shape and evolution of the quorum-sensing activation and cooperation landscape. As we showed in our work, even sub-MIC concentrations of Cm prevented the selection of the *mexT*^*−*^ allele and led to a reduction in the level of cooperativity (Fig. 4). It would be of interest to identify genetic mechanisms that help resolve this conflict when both antibiotic resistance and quorum-sensing directed cooperation are being selected for.

## Electronic supplementary material

Supplementary Tables

Supplementary Figure legends

Figure S1

Figure S2

Figure S3

Figure S4
