# Supplementary material for: Selection for increased quorum-sensing cooperation in Pseudomonas aeruginosa through the shut-down of a drug resistance pump
Source: ISME J. 2018 Jun 20;12(10):2458–69. doi: 10.1038/s41396-018-0205-y (PMC6154968; doi:10.1038/s41396-018-0205-y)
Supplement: Supplementary file 1 — Supplementary Tables [file 41396_2018_205_MOESM1_ESM.docx]

Table S1 - Bacterial strains and plasmids used in this study

| *Strain or Plasmid^a^* | *Relevant genotype^b^* | | *Reference* | *Lab No.* |
| --- | --- | --- | --- | --- |
| **Bacteria** |  | |  |  |
| *Pseudomonas aeruginosa* |  | |  |  |
| PAO1 | Wild-type (mexT^+^) (obtained from Greenberg Lab) | |  | AEA101 |
| PAO1 *mexT2* | Wild-type (*mexT*^C712A^) (obtained from Banin Lab) | | (Blus-Kadosh et al., 2013) | AEA325 |
| PAO1 *psdR1 mexT1* | PAO1 derivative; *psdR*^G41A^, *mexT* ^C983A^ | | This Study | AEA935 |
| PAO1 Δ*lasR* | PAO1 derivative; Δ*lasR*, unmarked deletion (obtained from Greenberg Lab) | | (Davies et al., 1998) | AEA102 |
| PAO1 Δ*lasR mexT2* | PAO1 *mexT2* derivative; Δ*lasR*, unmarked deletion | | This Study | AEA367 |
| PAO1 Δ*lasR* *psdR7* | PAO1 Δ*lasR* derivative; *psdR*^C209T^ | | This Study | AEA932 |
| PAO1 Δ*lasR* *psdR5 mexT6* | PAO1 Δ*lasR* derivative; *psdR*^G3A^, *mexT*^T722A^ | | This Study | AEA933 |
| PAO1 PT5 | Wild-type (mexT^-^) (obtained from Thilo Kohler) | | (Köhler et al., 2001) | AEA1174 |
| PAO1 PT149 | PT5 derivative; *nfxC*; overproduces MexEF-OprN (obtained from Thilo Kohler) | | (Köhler et al., 2001) | AEA1175 |
| PAO1 PT637 | PT149 derivative; *mexE*::ΩHg; does not produce MexEF-OprN (obtained from Thilo Kohler) | | (Köhler et al., 2001) | AEA1176 |
|  |  | |  |  |
| *Escherichia coli* DH12S | *mcrA Δ(mrr-hsdRMS-mcrBC) φ80d lacZΔM15 ΔlacX74 recA1 deoR Δ(ara, leu)7697 araD139 galU galK rpsL F' [proAB+ lacIqZΔM15]* | |  | AEC808 |
| **Plasmids** | |  |  |  |
| pPROBE-AT-GFP Cb^R^ | |  | This Study | AEC677 |
| pPROBE-P*_rhlA_*-GFP Cb^R^ | |  | This Study | AEC1154 |
| pPROBE-P*_rhlI_*-GFP Cb^R^ | |  | This Study | AEC1265 |
| pPROBE-P*_lasB_*-GFP Cb^R^ | |  | This Study | AEC654 |
| pPROBE-P*_mexE_*-GFP Cb^R^ | |  | This Study | AEC913 |
| pPROBE-P*_pqsA_*-GFP Cb^R^ | |  | This Study | AEC1263 |
| pMRP9-1 Cb^R^ | |  | (Davies et al., 1998) | AEC749 |
| pUB-BFP Cb^R^ | |  | This Study | AEC912 |
| pUB-GFP Cb^R^ | |  | This Study | AEC877 |
| pEX18-*lasR* Ap^R^, Cb^R^ | | Obtained from the Parsek lab |  | AEC845 |

^a^ Ap^R^, ampicillin resistant; Cb^R^, carbenicillin resistant.

^b^ Point mutations are marked as superscript after the relevant gene

|  | |  | Plasmid | | | | | | |
| --- | --- | --- | --- | --- | --- | --- | --- | --- | --- |
|  |  | pPROBE-AT | pPROBE-P*_rhlA_* | pPROBE-P*_rhlI_* | pPROBE-P*_lasB_* | pPROBE-P*_mexE_* | pPROBE-P*_pqsA_* | pUB-BFP | pUB-GFP |
| Strain | Wild-type (AEA101) | AEA181 | AEA250 | AEA760 | AEA180 | AEA459 | AEA1310 | AEA501 | AEA664 |
|  | *mexT2* (AEA325) | - | AEA329 | AEA764 | AEA327 | AEA460 | AEA1311 | AEA1268 | AEA505 |
|  | *psdR1; mexT1* (AEA935) | - | - | - | - | - | AEA1322 | AEA1266 | AEA1267 |
|  | Δ*lasR* (AEA102) | AEA144 | AEA251 | AEA770 | AEA145 | AEA466 | AEA1312 | AEA668 | AEA666 |
|  | Δ*lasR; mexT2* (AEA367) | AEA603 | AEA435 | AEA766 | AEA434 | AEA570 | AEA1313 | AEA473 | AEA506 |
|  | Δ*lasR; psdR7* (AEA932) | - | AEA631 | AEA768 | AEA629 | AEA639 | AEA1314 | AEA713 | AEA712 |
|  | Δ*lasR; psdR5; mexT6* (AEA933) | - | AEA358 | AEA772 | AEA356 | AEA465 | AEA1315 | AEA667 | AEA669 |
|  | PT5 (*mexT*^-^) (AEA1174) | - | AEA1179 | - | - | AEA1201 | AEA1304 | AEA1210 | AEA1211 |
|  | PT149 (*mexT*^+^) (AEA1175) | - | AEA1180 | - | - | AEA1202 | AEA1305 | AEA1205 | AEA1208 |
|  | PT637 (*mexT*^+^; Δ*mexE*) (AEA1176) | - | AEA1181 | - | - | AEA1203 | AEA1306 | AEA1212 | AEA1209 |

Table S2 - Transformed P. aeruginosa strains used in this study

Table S3 - Primers used in this study

| Primer | Sequence | |
| --- | --- | --- |
| *PpqsA -Fwd* | 5’-GCAAGCTTGCCCAGTGTACTACGCAATG-3’ | |
| *PpqsA -Rev* | 5’-GCGAATTCGACAGAACGTTCCCTCTTCAG-3’ | |
| *mexEF_*promoter_fw | 5’-ATGCAAGCTTTCTATTGATGCCGAACCTGCTGG-3’ | |
| *mexEF_*promoter_rv | 5’-ATATGAATTCGCTTGACTCCGCCAGTCGGTTTT-3’ | |
| *psdR*_seq_fwd | 5’-GGGTTCGGGTAGTTCATCTGTCA-3’ | |
| *psdR*_seq_rev | 5’-GATCGTCCGTTTGTTGTTGGTCG-3’ | |
| *mexT*_seq_fwd | 5’-GGGTGAATAACCTCATGGGT-3’ | |
| *mexT*_seq_rev | 5’-GATGGAATAAGCCGCACACC-3’ | |
| pA1-fwd | 5’-CGGAAGCGGAAGAGCGAAAATTTATCAAAAAGAGTG-3’ | |
| pA1-rev | 5’-GCGCGAATTCCTAGATGTGTGAAATTGTTATCC-3’ | |
| PstI-P*rhlA*-Fwd | 5’-GCCTGCAGCATCAAATCGGACAAGTG-3’ | |
| KpnI-P*rhlA*-Rev | 5’-GCGGTACCCTCACACCTCCCAAAAAT-3’ |  |
| P*rhlI* Fwd | 5’-TAAAGCTTCTGTTCGACCAGAGCCGGATG-3’ |  |
| *rhlI* pro rev | 5’-ATATGAATTCGACCAAGTCCCCGTGTCGTG-3’ |  |
| p*lasB* (S) | 5’-AGAGAAGCTTGCAGCAGCGGATCGTCGGCGA-3’ |  |
| p*lasB* (AS) | 5’-CACAGAGCTCGCCGCCTTGTGGGCTGA-3’ |  |

Supplementary References

Blus-Kadosh, I., Zilka, A., Yerushalmi, G. & Banin, E. 2013. The effect of pstS and phoB on quorum sensing and swarming motility in Pseudomonas aeruginosa. *PloS one,* 8**,** e74444.

Davies, D. G., Parsek, M. R., Pearson, J. P., Iglewski, B. H., Costerton, J. T. & Greenberg, E. 1998. The involvement of cell-to-cell signals in the development of a bacterial biofilm. *Science,* 280**,** 295-298.

Köhler, T., Van Delden, C., Curty, L. K., Hamzehpour, M. M. & Pechere, J.-C. 2001. Overexpression of the MexEF-OprN multidrug efflux system affects cell-to-cell signaling in Pseudomonas aeruginosa. *Journal of Bacteriology,* 183**,** 5213-5222.
