# Supplementary material for: Selection for increased quorum-sensing cooperation in Pseudomonas aeruginosa through the shut-down of a drug resistance pump
Source: ISME J. 2018 Jun 20;12(10):2458–69. doi: 10.1038/s41396-018-0205-y (PMC6154968; doi:10.1038/s41396-018-0205-y)
Supplement: Supplementary file 2 — Supplementary Figure legends [file 41396_2018_205_MOESM2_ESM.docx]

**Supplementary figure legends**

Fig. S1: Growth of lasR^+^ at high inoculum OD. Shown are growth factors of the three indicated genotypes used in Fig. 2C of the main text, when casein was inoculated at an optical density of 0.1. Compare with inoculation at optical density of 0.01 shown in Fig. 2C. Growth in this case seems to be limited by saturation of nutrients. Error bars represent the standard error of the mean.

Figure S2: Halo size on skim milk plates of the relevant genotypes. Shown are halos of colonies grown for 24 hours on skim milk plates (methods). Halo size is indicative of proteolytic activity.

Figure S3: Mean expression level per cell of a P*_pqsA_*-GFP transcriptional reporter after 24 hours of growth in 0.5% Casamino-acid medium for four relevant genotypes. Error bars represent the standard error of the mean. *** - p-value<10^-5^.

Figure S4: The effect of fluorescent reporters on relative fitness. Shown are three graphs of the relative fitness of a *mexT^-^* strain over a *mexT^+^* strain as a function of chloramphenicol concentration, as in Fig. 4 of the main text. The three graphs show the relative fitness results where the *mexT^-^* strain carries a BFP plasmid reporter (blue) or a GFP plasmid reporter (green). The black line shows the average behavior, shown also in Fig. 4. Error bars represent the standard error of the mean.
