# Supplementary figures and images for: Selection for increased quorum-sensing cooperation in Pseudomonas aeruginosa through the shut-down of a drug resistance pump
Source: ISME J. 2018 Jun 20;12(10):2458–69. doi: 10.1038/s41396-018-0205-y (PMC6154968; doi:10.1038/s41396-018-0205-y)

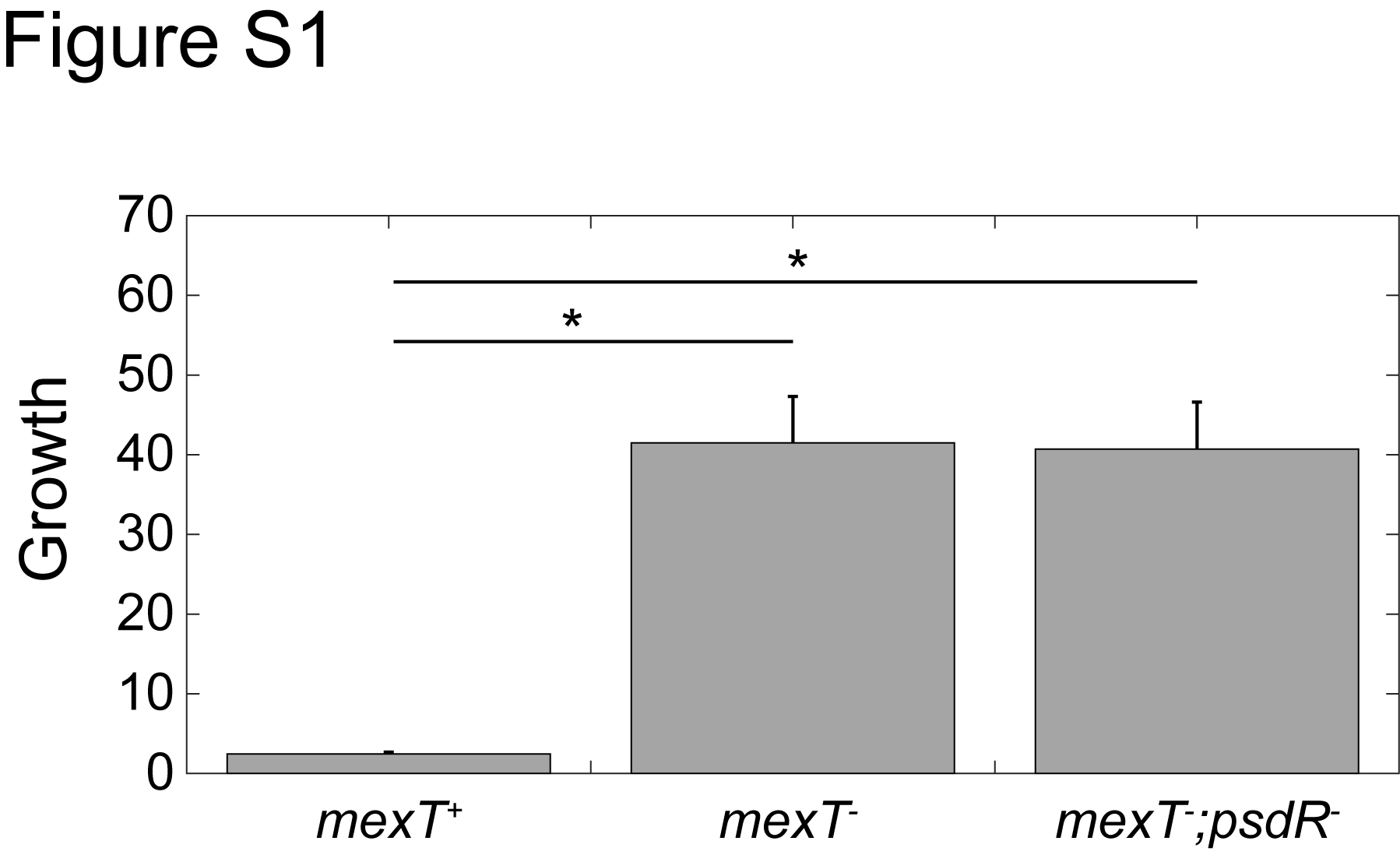

Supplement: Supplementary file 3 — Figure S1 [file 41396_2018_205_MOESM3_ESM.tif]

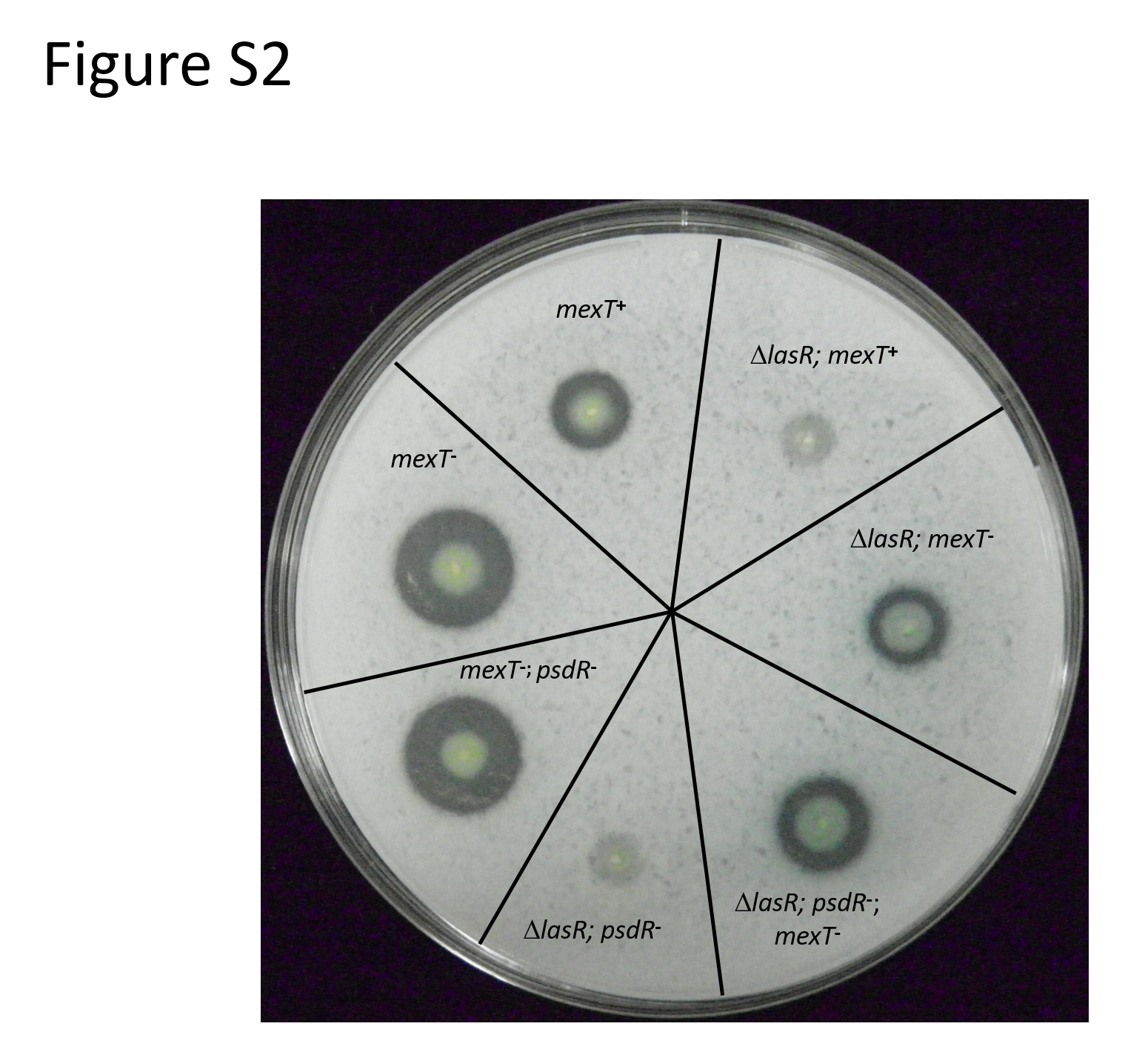

Supplement: Supplementary file 4 — Figure S2 [file 41396_2018_205_MOESM4_ESM.tif]

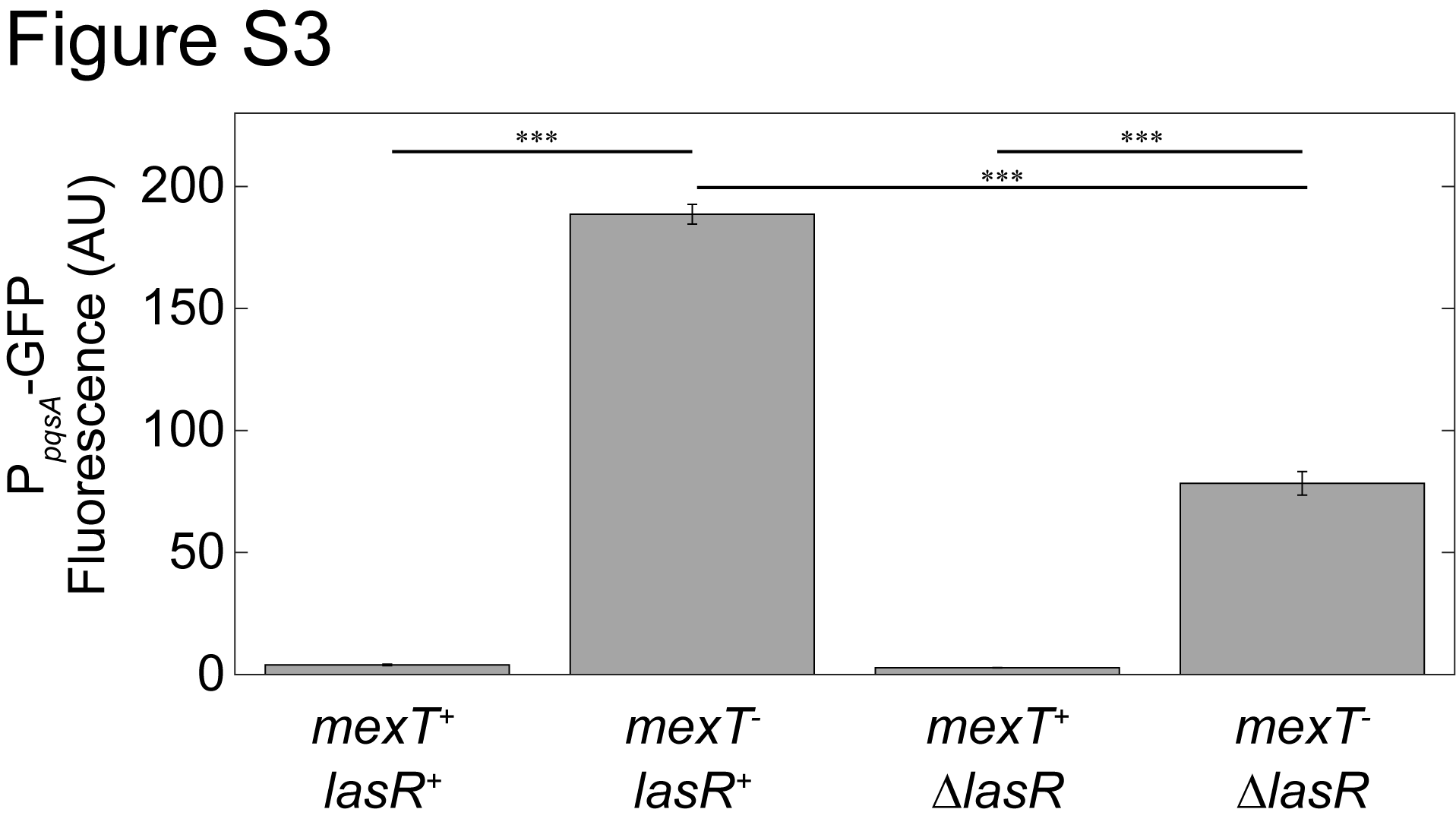

Supplement: Supplementary file 5 — Figure S3 [file 41396_2018_205_MOESM5_ESM.tif]

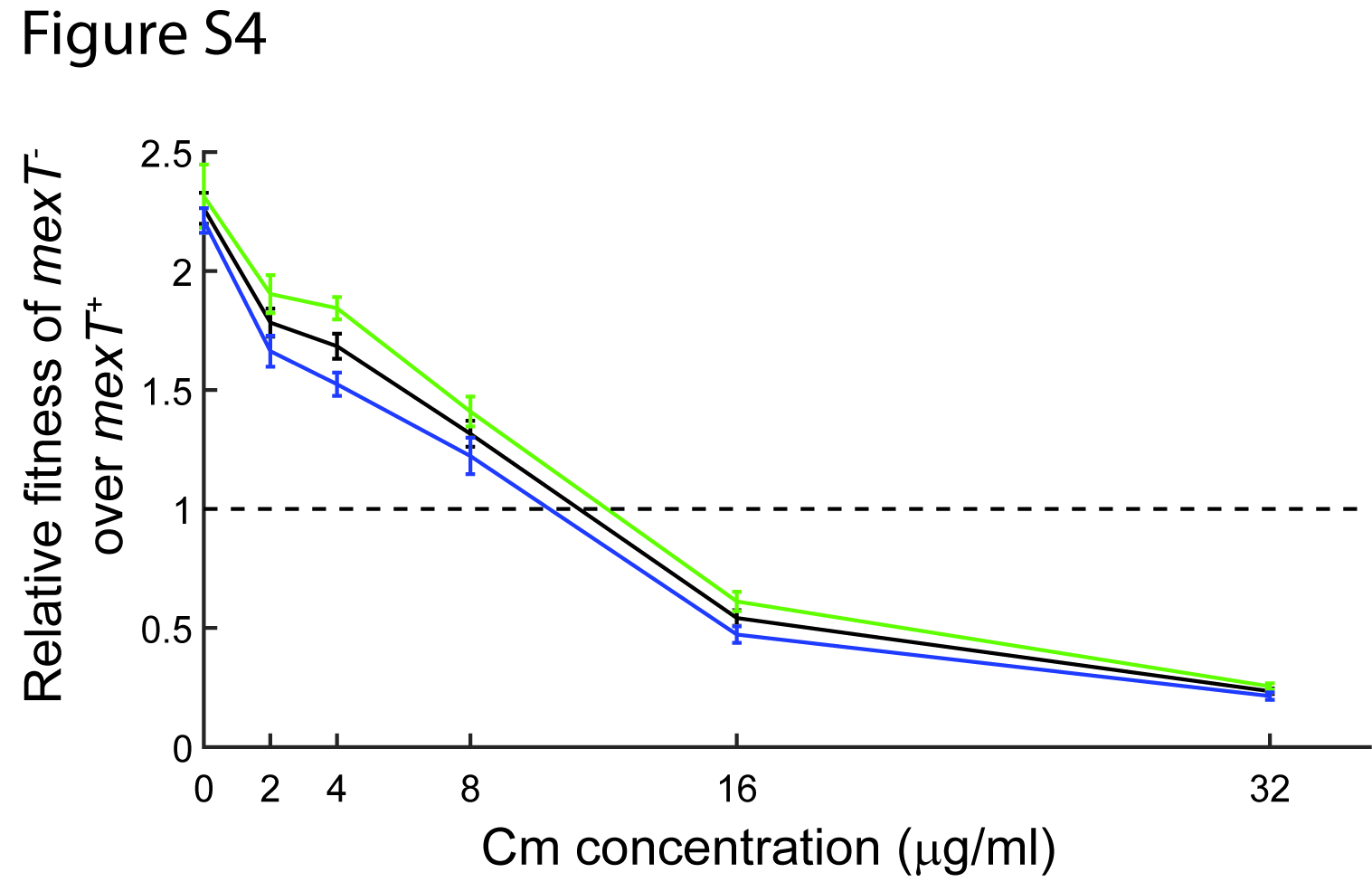

Supplement: Supplementary file 6 — Figure S4 [file 41396_2018_205_MOESM6_ESM.tif]
